# Anabolic-androgenic steroids and brain injury: miRNA evaluation in users compared to cocaine abusers and elderly people

**DOI:** 10.18632/aging.103512

**Published:** 2020-08-03

**Authors:** Francesco Sessa, Monica Salerno, Luigi Cipolloni, Giuseppe Bertozzi, Giovanni Messina, Giulio Di Mizio, Alessio Asmundo, Cristoforo Pomara

**Affiliations:** 1 Department of Clinical and Experimental Medicine, University of Foggia, Foggia 71122, Italy; 2 Department of Medical, Surgical and Advanced Technologies “G.F. Ingrassia”, University of Catania, Catania 95121, Italy; 3 Department of Legal, Historical, Economic and Social Sciences, University of Catanzaro, Catanzaro 88100, Italy; 4 Department of Biomedical and Dental Sciences, and of Morphological and Functional Images, Section of Legal Medicine, University of Messina, Messina 98121, Italy

**Keywords:** anabolic androgenic steroids, nervous systems, cocaine, aging, brain damage

## Abstract

Anabolic-androgenic steroids (AASs) can be used to treat both hormonal diseases and other pathologies characterized by muscle loss (aging, cancer, and AIDS). Even if the adverse effects related to the misuse of AASs have been well studied in different systems and apparatuses, knowledge about brain damage is poor.

In this scenario, this experimental study aimed to analyze the role of several microRNAs (miRNAs) in brain damage after AAS misuse, to better comprehend the underlying mechanisms. The research hypothesis at the base of this experimental study is that the chronic use of AASs may be associated to brain damage with a dysregulation of these miRNAs. Moreover, miRNA expression values were compared among three different groups, “AAS” group, “Cocaine” group and “Aging” group, in order to define if AAS brain damage can be compared with the brain impairment linked to aging and/or cocaine assumption.

This experimental study revealed that the tested miRNAs (hsa-miR-21-5p, hsa-miR-34a-5p, hsa-miR-124-5p, hsa-miR-132-3p, and hsa-miR-144-3p) were overexpressed in all enrolled groups. In the light of the presented results, the identification of specific circulating and/or tissue biomarkers is challenging for the scientific community. Further studies with larger samples are needed to confirm these interesting findings.

## INTRODUCTION

Anabolic-Androgenic Steroids (AASs) are considered synthetic variations of human testosterone. The legitimate use of these kinds of drugs is strictly linked to different human hormonal diseases, such as delayed puberty [1–5]. Moreover, steroids can be used to treat conditions that cause muscle loss: for example, aging, cancer, and AIDS [6, 7].

Considering their anabolic effect, these kinds of drugs are often abused by athletes or individuals with the goal of improving their physical appearance. The long-term abuse of AASs can lead to serious health consequences [8], generating different human hormonal diseases such as severe acne, development of breasts in men (gynecomastia) [9], facial and body hair growth in women (hirsutism) [10], shrinkage of testicles [10–12], baldness in both sexes, irregular menstruation, and infertility [13]; moreover, AAS abuse may generate yellowing of the eyes or skin (jaundice), high blood pressure [14], and liver tumors or other cancers [15, 16].

Even if the adverse effects related to the misuse of AASs have been well studied in different systems and apparatuses, our knowledge about brain damage is poor [17]. Several psychological adverse effects are frequently reported in AAS users, such as dramatic mood changes (“roid rages”) and maniac behavior [18, 19], irritability [20], and anxiety [21]. Moreover, other studies described memory impairment and cognitive deficits in long-term AAS users [22, 23]. Despite these biological effects, the mechanisms of action at the brain level are not fully known. Nevertheless, two mechanisms of action are hypothesized: the first is the well-known one of the pro-apoptotic activity exerted by AASs; the other is that AAS abuse generates cerebral atrophy, reducing the cortical volume and thinning the cortex, even if further studies are needed to better ascertain this mechanism [17]. On the one hand, the pivotal role of programmed cell death (apoptosis) in age-related diseases has been well outlined for different organs (particularly brain, immune system, heart, and reproductive systems) [24–26]. On the other hand, recreational AAS users have combined the assumption of anabolic substances with other substances, such as cocaine, methamphetamine, and smart drugs. In fact, Sagoe et al. reported in their review important evidence to support the associations between AAS use and the use of a wide range of other licit and illicit substances among users [27]. Obviously, the practice of mixing two or more substances amplifies the risk of negative drug interactions, worsening each adverse effect [28]. The result of this interaction can include several different consequences, such as an enhancement of the adverse effects of each drug, serious damage to different organs, and serious neurological or psychological complications [29, 30]. It has been well described that AAS assumption generates an alteration in amygdala functions, with subsequent neurochemical abnormalities and visuospatial memory impairment [31].

**Figure 1:**
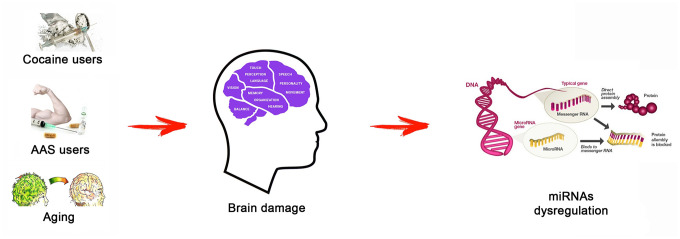
**The research hypothesis at the base of this research study: different status (AAS use, cocaine use, and aging) may be associated to brain damage, generating a miRNA dysregulation.** Testing the expression values of several miRNAs involved in important brain functions (miR-21, miR-34, miR-124, miR-132, and miR-144), this study aimed highlighting the differences of these molecular biomarkers among the three tested groups.

In this scenario, this experimental study aimed to analyze the role of several microRNAs (miRNAs) in brain damage after AAS misuse, to better understand the underlying mechanisms. Considering that previous studies have demonstrated that specific miRNAs (miR-21, miR-34, miR-124, miR-132, and miR-144) play a pivotal role in the control of important target genes involved both in neuronal apoptosis and neuronal stress-induced adaptation [32–37], the research hypothesis at the base of this experimental study was that the chronic use of AASs may be associated to brain damage with a dysregulation of these miRNAs. Moreover, miRNA expression values were compared among three different groups, “AAS” group, “Cocaine” group and “Aging” group, in order to define if AAS brain damage can be compared with the brain impairment linked to aging and/or cocaine assumption (Figure 1).

## RESULTS

The quantitative analysis evaluated the expression levels of the following miRNAs: hsa-miR-21-5p, hsa-miR-34a-5p, hsa-miR-124-5p, hsa-miR-132-3p, and hsa-miR-144-3p, in the three groups: “AAS” group, “Cocaine” group, and “Aging” group. The values of the “Control” group were used to apply the ΔΔCT method, as indicated in Material and Methods. In Table 1 the results of the expression values of each miRNA tested, subdivided for each group are summarized.

**Table 1:** Expression levels of miRNAs analyzed in each group.

**GROUPS**	**Mean Expression levels (endogenous control: miR-186)**				
	**miR-21-5p**	**miR-34a-5p**	**miR-124-5p**	**miR-132-3p**	**miR-144-3p**
**AAS (Mean values)**	1.58 ± 0.3	11.26 ± 1.21	1.18 ± 0.34	8.09 ± 3.3	1.39 ± 0.25
**Cocaine (Mean values)**	0.86 ± 0.17	12.33 ± 1.84	0.4 ± 0.25	8.74 ± 3.51	0.59 ± 0.3
**Aging (Mean values)**	1.9 ± 0.5	2.16 ± 0.91	0.05 ± 0.04	1.11 ± 0.57	1.07 ± 0.17

Moreover, a statistical analysis was performed analyzing the expression values of each miRNA tested, comparing all groups.

**Figure 2:**
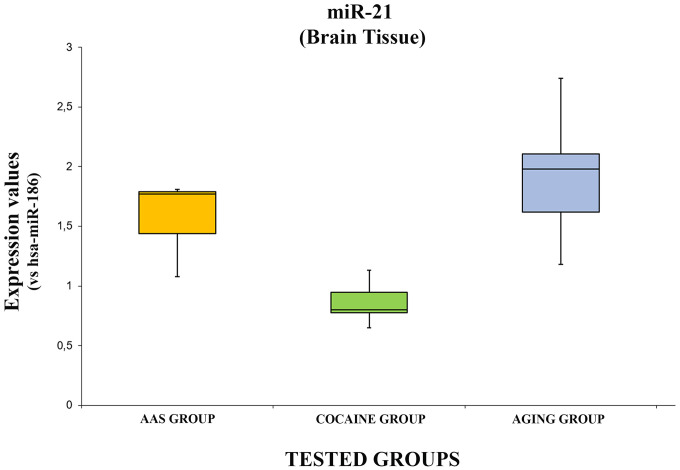
**In this figure, the box plot analyses compare the expression levels of hsa-miR-21-5p (endogenous control miR-186) in each group.**

The expression values of hsa-miR-21-5p are summarized with box plot analyses in Figure 2.

There were statistically significant differences among groups as determined by one-way ANOVA [F(2.18) = 3.55, p = <0.05(0.0001)]. Moreover, the expression level of hsa-miR-21-5p was significantly higher in the “AAS” group compared with the “Cocaine” group [F (1.12) = 4.74, p = <0.05 (0.00013)], while no statistical differences were noted comparing the “AAS” group with the “Aging” group [F (1.12) = 4.74, p = >0.05(0.18)]. Finally, the expression values of this miRNA were significantly higher in the “Aging” group compared with the “Cocaine” group [F(1.12) = 4.74, p = <0.05 (0.0002)].

**Figure 3:**
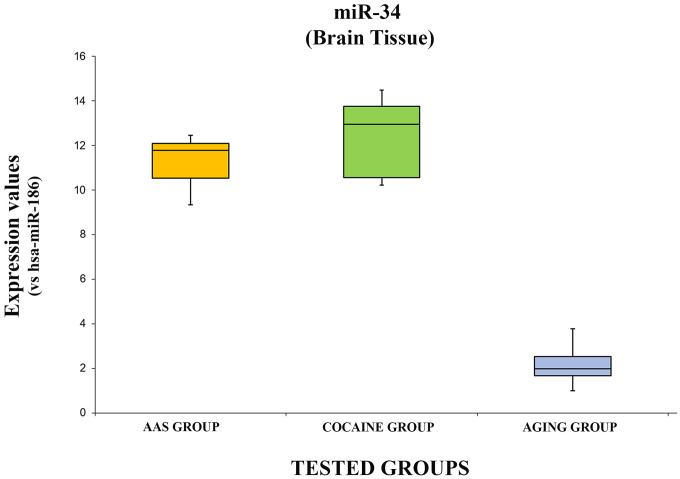
**In this figure, the box plot analyses compare the expression levels of hsa-miR-34a-5p (endogenous control miR-186) in each group.**

The expression values of hsa-miR-34a-5p are summarized with box plot analyses in Figure 3.

There were statistically significant differences among the groups as determined by one-way ANOVA [F(2.18) = 3.55, p = <0.05(5.74x10^-11^)]. Moreover, the expression level of hsa-miR-34a-5p was significantly higher in the “AAS” group compared with the “Aging” group [F (1.12) = 4.74, p = <0.05 (2.14x10^-9^)], while no statistical differences were noted comparing the “AAS” group with the “Cocaine” group [F (1.12) = 4.74, p = >0.05(0.22)]. Finally, the expression values of this miRNA were significantly higher in the “Cocaine” group compared with the “Aging” group [F(1.12) = 4.74, p = <0.05 (1.86x10^-8^)].

**Figure 4:**
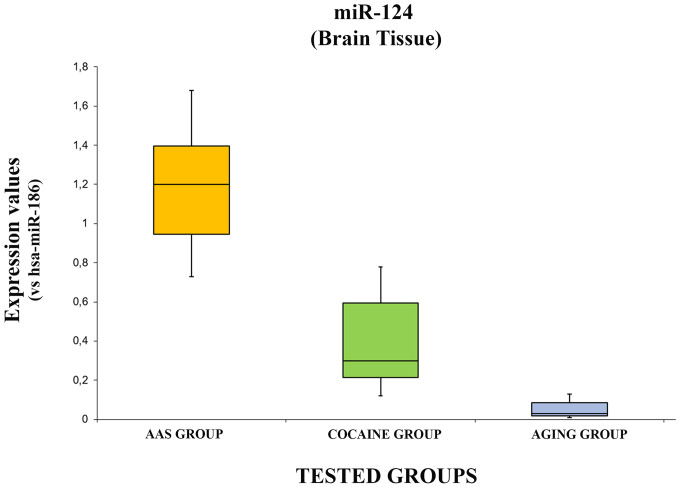
**In this figure, the box plot analyses compare the expression levels of hsa-miR-124-5p (endogenous control miR-186) in each group.**

The data concerning the expression levels of hsa-miR-124-5p are summarized in Figure 4.

There were statistically significant differences among groups as determined by one-way ANOVA [F(2.18) = 3.55, p = <0.05(4.37x10^-7^)]. Moreover, the expression level of hsa-miR-124-5p was significantly higher in the “AAS” group both compared with the “Aging” group [F (1.12) = 4.74, p = <0.05 (2.08x10^-6^)] and with the “Cocaine” group [F (1.12) = 4.74, p = >0.05(0.0004)]. Finally, the expression values of this miRNA were significantly higher in the “Cocaine” group compared with the “Aging” group [F(1.12) = 4.74, p = <0.05 (0.004)].

**Figure 5:**
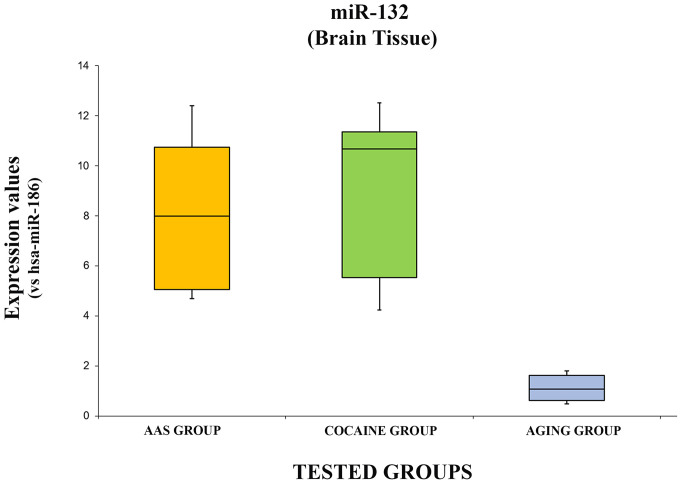
**In this figure, the box plot analyses compare the expression levels of hsa-miR-132-3p (endogenous control miR-186) in each group.**

The expression levels of hsa-miR-132-3p are summarized with the box plot analyses in Figure 5.

There were statistically significant differences among groups as determined by one-way ANOVA [F(2.18) = 3.55, p = <0.05(0.0001)]. Moreover, the expression level of hsa-miR-132-3p was significantly higher in the “AAS” group compared with the “Aging” group [F (1.12) = 4.74, p = <0.05 (0.0001)], while no statistical differences were reported compared with the “Cocaine” group [F (1.12) = 4.74, p = >0.05(0.73)]. Finally, the expression values of this miRNA were significantly higher in the “Cocaine” group compared with the “Aging” group [F(1.12) = 4.74, p = <0.05 (0.0001)].

**Figure 6:**
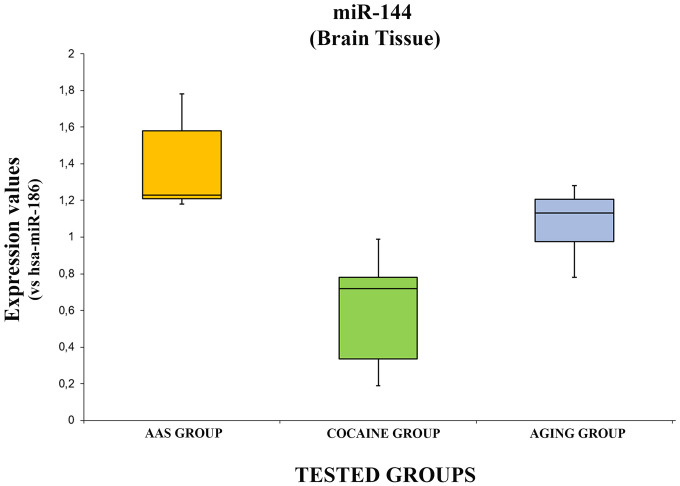
**In this figure, the box plot analyses compare the expression levels of hsa-miR-144-3p (endogenous control miR-186) in each group.**

The expression values of hsa-miR-144-3p are summarized with box plot analyses in Figure 6.

There were statistically significant differences among groups as determined by one-way ANOVA [F(2.18) = 3.55, p = <0.05(10x4.93^-5^)]. Moreover, comparing the expression level of the hsa-miR-144-3p singularly, it was significantly higher in the “AAS” group compared both with the “Cocaine” [F (1.12) = 4.74, p = <0.05 (0.0001)] and the “Aging” groups [F (1.12) = 4.74, p = <0.05(0.01)]. Moreover, the expression values of this miRNA in the “Aging” group was significantly higher compared with the “Cocaine” group [F(1.12) = 4.74, p = <0.05 (0.003)].

## DISCUSSION

The present study demonstrated that the different investigated situations, AAS assumption, cocaine abuse and aging, were associated with miRNA dysregulation at the brain level. Particularly, the tested miRNAs (miRNA hsa-miR-21-5p, miR-34, hsa-miR-124-5p, hsa-miR-132-3p, and miR-144) were overexpressed in all tested groups (“AAS”, “Cocaine”, and “Aging” groups) compared with the control. In the light of the these results the identification of specific circulating and/or tissue biomarkers is challenging for the scientific community [38]. In the forensic field the identification of new molecular biomarkers strictly related to specific brain damage could be very useful to define the exact cause of death; in fact, miRNAs are very stable without changes generated by postmortem modifications. Nevertheless, this kind of biomarkers could be very useful in the near future for anti-doping purposes, or to identify brain damage linked to aging.

Different studies have reported an important role in the anti-inflammatory systems for miR-21: it has been reported that it has an important effect in cases of brain-injury, alleviating leakage of the microvascular endothelial barrier through suppression of inflammation and apoptosis [39]. Moreover, it has been described that it is overexpressed in damaged tissue compared with healthy control tissue, suggesting a role as a general biomarker of inflammation-associated diseases [40]. Furthermore, this miRNA is involved in mood control: it has been described dysregulated in several stress situations [41] and bipolar disorders [42]. Finally, it is very important to highlight that this miRNA has been found overexpressed in the brain after a lesion [43]. Moreover, an *in vivo* study described that the basal expression levels of this miRNA in aging were significantly higher compared with healthy adults [44]. In agreement with these studies, in the present research, the levels of miR-21 were overexpressed in all tested groups, even if it was significantly higher both in the “AAS” and the “Aging” groups. It is clear that all analyzed conditions (AAS and cocaine assumption, and aging) generate brain damage with a miRNA dysregulation. The expression levels suggest a role for this miRNA in anti-inflammatory and antiapoptotic actions, particularly in response to brain damage generated by AAS misuse and aging. Moreover, in the light of the present results, miR-21 could be considered both a promising marker for brain damage and a therapeutic target in brain damage evaluation [45].

miR-34 levels were overexpressed in all groups, even if they were significantly higher in the “AAS” and the “Cocaine” groups; it is important to note that there were no significant differences between the “AAS” and the “Cocaine” groups, highlighting a similar role for this miRNA in brain damage linked to the assumption of cocaine and anabolic-androgenic substances. Several studies performed on animal models have described an essential role for this miRNA in the modulation of cocaine intake: notably, it has been described overexpressed in mice exposed to acute restraint stress and repeated social defeat stress. In the same study, Doura and Unterwald described that the overexpression of miR-34 in amygdala tissue represents a physiological reaction to stressful situations, reducing anxiogenic effects [46]. Other studies reported that miR-34 is involved in neuronal pro-apoptotic mechanisms: in this way, it is commonly thought that therapies targeting miR-34 may be beneficial as novel therapies for neurodegenerative diseases and related central nervous system diseases. A therapy against this miRNA could be very important in several diseases that occur in a prevalent manner in elderly people, such as Parkinson’s disease (PD), and Alzheimer’s disease (AD) [47]. A more recent study performed through neuroimaging has highlighted that subjects who used AASs had a thinner cortex, particularly in pre-frontal regions: this area is involved in inhibitory control and emotional control [48]. In this regard, it has been reported that the upregulation of miR-34 has been described in the temporal cortex of post-mortem AD brain tissue. Moreover, overexpression of miR-34a in primary neurons can generate mitochondrial dysfunction, acting in a pro-apoptotic way; finally, the upregulation of miR-34 can be linked to a reduction in glucose metabolism within the hippocampus, promoting the cell damage frequently found in AD patients [49]. In consideration of these findings, our results are in line with previous studies: miR-34 was overexpressed in all tested groups, even if in the “Aging” group the levels were lower compared with the other groups. No significant differences were found evaluating the expression levels between the “AAS” and the “Cocaine” groups. The differences in the expression values among the tested groups could be linked to the different conditions at the brain tissue level. Indeed, neurodegenerative diseases are commonly found in the brain tissue of the elderly, while both the “AAS” and the “Cocaine” groups were made up of young subjects who showed a higher reaction to the stressful condition of the brain damage generated both by cocaine and the anabolic-androgenic agent assumption.

miR-124 is a very promising biomarker for brain tissue diseases because it is well known that this miRNA is widely expressed in different areas of the brain with the exception of the pituitary gland [50]. Several studies, performed in animal models, confirmed a pivotal role for this miRNA: notably, it plays an essential role in the regulation of signaling molecules improving synaptic plasticity [51, 52]. For these reasons, it is commonly thought that it has been involved in the response to several neurodegenerative diseases such as AD and PD, improving brain function. This miRNA is involved in the response to cocaine assumption improving brain tissue plasticity [53]. Moreover, it seems to be involved in the complex mechanisms that regulate the response to stress situations. In this scenario, the expression levels of this miRNA can be very important in the determination of alternative splicing in the brain, modifying cholinergic neuro-transmission [54, 55]. In this regard, it is very interesting to note that several studies suggested that a reduction of miR-124 expression through a particular diet regimen could positively influence the orexinergic and serotoninergic systems [56–60]. The results of the present study demonstrated that this miRNA is overexpressed in the “AAS” group compared with both the “Cocaine” and the “Aging” groups. These findings are in agreement with a previous study that reported that the overexpression of miR-124 may improve functional recovery, reducing lesion volume and suppressing apoptosis in rats after brain injury [61]. Obviously, young subjects are more responsive compared with elderly people: for these reasons the expression levels of this miRNA are higher in the subjects of the “AAS” and the “Cocaine” groups. Finally, the “Aging” group has the lowest expression levels: these results should be linked to the reduction in brain plasticity detected in this group.

miR-132 is involved in several diseases such as neural differentiation, neural growth and migration and neural plasticity [62]. This miRNA is very important in the regulation of degenerative effects linked to aging: indeed, Hadar et al. described a negative correlation between the expression levels of this miRNA and aging, while they reported high levels in subjects with AD [63]. Moreover, as reported in previous studies, miR-132 is up-regulated in several diseases such as multiple sclerosis, PD, several infections, epilepsy, disturbance of consciousness, memory disturbance, and schizophrenia, while it has been reported as being down-regulated in AD, progressive supranuclear palsy, and depression [62]. Obviously, all these findings should be confirmed with further studies. The results of the present experimental study show that the expression levels of miR-132 were significantly higher in the “AAS” and the “Cocaine” groups compared with the “Aging” group. These results suggest that in these groups there is a cellular activity finalized to the reduction of adverse effects linked to the use of these substances (androgen and cocaine). Moreover, considering that the “Aging” group showed the worst expression levels, it could be thought that this kind of reaction does not occur in the brain tissue of the elderly: in fact, both groups are made up of young subjects who are more reactive to stress conditions. Moreover, it could be hypothesized that the oxidative stress condition generated by AAS assumption is about the same as that induced by cocaine use.

In the present study the expression levels of miR-144 were overexpressed in all groups compared with the control, even if it was significantly higher in the “AAS” group. miR-144 was previously described as a promising biomarker in cocaine abuse [64, 65]. It was described that the aberrant expression of this miRNA is related with both the chronic use of cocaine and with the release phase of metabolites [65]. Moreover, miR-144 plays a pivotal role in aging-related brain damage. Indeed, in several studies it has been described that the expression levels of this miRNA is upregulated in aging subjects, suggesting a coordinating role in the post-transcriptional regulation of a group of genes involved in brain aging control. Moreover, it is important because it regulates responsiveness to environmental signals [66–68]. The mechanism of action of brain system impairment is related to β-amyloid accumulation. Particularly, it has been reported that after traumatic brain injury, miR-144 is overexpressed, suppressing ADAM10 expression [64, 69]. Furthermore, considering that AAS assumption is strictly linked with different behavior modifications, an important regulation role for this miRNA has been described, controlling the expression of the metabotropic glutamate receptor gene GRM7, which is involved in mood disorders and attention deficit hyperactive disorder [70, 71].

It is important to note that this pilot study was conducted on tissues sampled during autopsy. This evidence represents the strengths of this study. Thanks to the post mortem findings, all groups were composed of subjects deceased due to an exact cause of death. Particularly, referring to the “AAS” and “Cocaine” groups, we enrolled subjects that certainly had used only the respective substance, with negative toxicological tests for other illicit drugs. Moreover, we compared the expression values of the selected miRNAs with the data obtained from the control group that was composed of healthy subjects who had died due to traumas.

The main limitation of this study is related to the fact that even if the androgen-androgenic or cocaine use was ascertained through a toxicological examination, data about the exact duration of use is unknown. Another significant limitation is related to the small number of subjects enrolled in each group. Concerning this last consideration, it is important to highlight that the number of subjects who die with a positive toxicological test for anabolic-androgenic steroids is not very high in Italy: for example, in our Institute, we were able to select only 7 cases analyzing about 1700 autopsy records.

## CONCLUSIONS

Nowadays, the use of miRNAs as bio-signatures of several diseases represents an essential tool of experimental studies. Their role has been mainly investigated from 2007 with the aim of identifying new molecular biomarkers in the field of cancer research. In the last few years, thanks to modern technologies, this kind of approach has become very promising in all fields of medicine [72–74]. To date, miRNA expression dosage has become a routine practice in different clinical applications, such as viral infection diagnosis, cancer characterization, and cardiovascular disorder diagnosis. Moreover, these biomarkers are becoming very important in brain injury research [69].

The research to better understand the mechanisms underlying brain systems is very challenging: miRNAs are very important both in neurological activity and in behavior control. The use of different drugs is considered one of the most important problems in developed countries. Today, thanks to the online market, the use of several substances is growing, particularly in young people both to improve physical performance and for an aesthetic image.

Another important problem for developed countries is the increasing number of elderly people. Moreover, an aged-population has different health problems related to neurodegenerative diseases at the brain level. Notably, these kinds of problems generate a high cost for the public health services of these countries.

In this scenario, this experimental study aimed to analyze the role of several miRNAs in brain damage after AAS misuse, to better understand the underlying regulation mechanisms. Moreover, miRNA investigation was performed in three groups: “AAS” users, “Cocaine” abusers and “Aging” people. The results highlighted that the investigated miRNAs presented different expression patterns in consideration of the tested diseases, suggesting a pivotal role. Moreover, this study could be considered very important to better define the mechanisms of action at the brain level. To date, few studies have been performed in this way: little is known, particularly concerning adverse effects exerted at the brain level by AAS assumption. As described above, miR-34 and miR-132 were significantly higher both in the “AAS” and the “Cocaine” groups compared with the “Aging” group, suggesting a pivotal role in adverse brain injury effects. Moreover, this study represents a pilot study to define if these expression patterns could be studied in other biological samples (plasma, urine) in subjects with different brain injury linked to aging, AAS and/or cocaine abuse, to identify reliable biomarkers that could be applied in clinical practice. miR-34 and miR-132 were expressed similarly in both the “AAS” and the “Cocaine” groups, more than 11/12 times compared with controls: for these reasons, they could be considered good candidates to define molecular biomarkers of brain damage.

Another important consideration is related to the diffused practice of combining AAS use with other psychoactive substances, such as cocaine: in this condition, it has been well described that the level of aggression increases, generating mental disorders such as hypomania and depression [75]. Finally, both miRNAs could be significantly overexpressed in a subject who combined the use of anabolic-androgenic substances with cocaine.

In the light of these findings, further studies with larger samples are needed to confirm these interesting findings.

## MATERIALS AND METHODS

### Selected cases

All samples were selected analyzing the autopsy documentation of all cases examined by the Institute of Legal Medicine of Foggia from 2001 to September 2019 (about 1700 autopsies). All procedures were performed in accordance with the Declaration of Helsinki and were approved by the Scientific Committee of the University of Foggia.

Seven cases of young men (29 - 40 years), with a toxicological positive test for anabolic-androgenic agents, were selected (mean age 33.28 ± 4.68 years; mean body mass index (BMI) 27.04 ± 1.07). These samples made up the “AAS” group.

Seven cases of men who had died with a toxicological test positive for drug abuse (cocaine) were selected (mean age 29.42 ± 6.1 years; mean BMI 23.25 ± 1.67), forming the “Cocaine” group. Seven cases of older men who died of sudden cardiac arrest (mean age 77.28 ± 3.25 years; mean BMI 23.7 ± 1.4), making up the “Aging” group.

Finally, seven cases of healthy men without neurological diseases (mean age 44.85 ± 5.75 years; mean BMI 26.5 ± 1.88), who had died in car accidents were selected as reference samples in the Real-Time PCR reactions (“Control” group).

### miRNA selection

The databases Medline, Scopus, Web of Science, and Google Scholar were searched from January 2007 to December 2019, using the following keywords: “Brain Injury”, “Brain Damage”, “miRNA”, “miRNA dysregulation”, “Aging”, “Cocaine”, and “Anabolic-Androgenic Steroids”. The main keywords, “Brain Injury” and “miRNA”, were searched for in association with each of the others.

At the end of the literature review, the following miRNAs were selected: hsa-miR-21-5p, hsa-miR-34a-5p, hsa-miR-124-5p, hsa-miR-132-3p, and hsa-miR-144-3p.

### miRNA quantitative real-time PCR (qRT-PCR)

The Recover All Total Nucleic Acid Isolation Kit (Life Technologies) was used to obtain total RNA working from formaldehyde-fixed paraffin-embedded (FFPE) brain samples (four 20 μm sections) as previously described [32]. To quantify the obtained RNA, the Qubit Fluorometer with the Qubit RNA HS Assay Kit (Life Technologies) was used.

To obtain the miRNA profiling of the selected miRNAs (hsa-miR-21-5p, hsa-miR-34a-5p, hsa-miR-124-5p, hsa-miR-132-3p, and hsa-miR-144-3p) the TaqMan Advanced miRNA Assay (ThermoFisher Scientific) kit was used. This kit was composed of a pre-formulated primer and probe set. Following the protocol kit cDNA was obtained, running the samples in the StepOnePlus Real-Time PCR System (ThermoFisher Scientific), analyzing the raw data with the relative software (version 2.3). The expression value data were analyzed following the method of the normalization with the endogenous control: for this experimental study, miR-186-5p (TaqMan Advanced miRNA Assays, ThermoScientific) was used as the endogenous control. Normalization to endogenous control genes is currently the most accurate method to correct for potential biases that are caused by sample collection, variation in the amount of starting material, RT efficiency, and RNA preparation and quality.

Expression fold changes were computed using the 2^−ΔΔCt^ calculation [76], where ΔCt = Ct (test miRNA) – Ct (mir-186-5p) and ΔΔCt = ΔCt (individual sample) - ΔCt (control median samples).

### Statistics

Descriptive statistical analyses were performed using different software packages (Microsoft Office Excel 2007, StataIC, StataCorp and R. To compare the data among the tested groups the one-way ANOVA (the aov function in R) with post-hoc pair wise comparisons was performed with Tukey’s Honestly Significant Difference (the TukeyHSDfunction in R). P < 0.05 was considered to represent a statistically significant difference.
